# Water level affects availability of optimal feeding habitats for threatened migratory waterbirds

**DOI:** 10.1002/ece3.3566

**Published:** 2017-11-07

**Authors:** Yaara Aharon‐Rotman, John McEvoy, Zheng Zhaoju, Hui Yu, Xin Wang, Yali Si, Zhenggang Xu, Zeng Yuan, Wooseog Jeong, Lei Cao, Anthony D. Fox

**Affiliations:** ^1^ State Key Laboratory of Urban and Regional Ecology Research Center for Eco‐Environmental Sciences Chinese Academy of Sciences Beijing China; ^2^ Conservation Ecology Centre National Zoological Park Smithsonian Conservation Biology Institute Front Royal VA USA; ^3^ Key Laboratoryof Digital Earth Science Institute of Remote Sensing and Digital Earth Chinese Academy of Sciences Beijing China; ^4^ Ministry of Education Key Laboratory for Earth System Modeling Department of Earth System Science Tsinghua University Beijing China; ^5^ Resource Ecology Group Wageningen Universit Wageningen The Netherlands; ^6^ Key Laboratory of Forestry Remote Sensing Based Big Data Ecological Security for Hunan Province Central South University of Forestry and Technology Changsha China; ^7^ Animal and Plant Quarantine Agency Gimcheon‐si, Gyeongsangbuk‐do Korea; ^8^ University of Chinese Academy of Sciences Shijingshan District, Beijing China; ^9^ Department of Bioscience Aarhus University Rønde Denmark

**Keywords:** *Anser albifrons*, *Anser cygnoides*, foraging ecology, geese, grassland, habitat selection, inundation, Poyang Lake, water table recession, Yangtze River

## Abstract

Extensive ephemeral wetlands at Poyang Lake, created by dramatic seasonal changes in water level, constitute the main wintering site for migratory Anatidae in China. Reductions in wetland area during the last 15 years have led to proposals to build a Poyang Dam to retain high winter water levels within the lake. Changing the natural hydrological system will affect waterbirds dependent on water level changes for food availability and accessibility. We tracked two goose species with different feeding behaviors (greater white‐fronted geese *Anser albifrons* [grazing species] and swan geese *Anser cygnoides* [tuber‐feeding species]) during two winters with contrasting water levels (continuous recession in 2015; sustained high water in 2016, similar to those predicted post‐Poyang Dam), investigating the effects of water level change on their habitat selection based on vegetation and elevation. In 2015, white‐fronted geese extensively exploited sequentially created mudflats, feeding on short nutritious graminoid swards, while swan geese excavated substrates along the water edge for tubers. This critical dynamic ecotone successively exposes subaquatic food and supports early‐stage graminoid growth during water level recession. During sustained high water levels in 2016, both species selected mudflats, but also to a greater degree of habitats with longer established seasonal graminoid swards because access to tubers and new graminoid growth was restricted under high‐water conditions. Longer established graminoid swards offer less energetically profitable forage for both species. Substantial reduction in suitable habitat and confinement to less profitable forage by higher water levels is likely to reduce the ability of geese to accumulate sufficient fat stores for migration, with potential carryover effects on subsequent survival and reproduction. Our results suggest that high water levels in Poyang Lake should be retained during summer, but permitted to gradually recede, exposing new areas throughout winter to provide access for waterbirds from all feeding guilds.

## 1 INTRODUCTION

The extensive and numerous ephemeral wetlands of the Yangtze River floodplain (YRf) are ecologically and economically important. Recharged by summer monsoonal rains, the river elevates water levels and brings nutrients and sediments, but in autumn and winter, water levels drop, exposing the largest concentration of shallow and seasonal flooded wetlands in the world (de Leeuw et al., 2006). These wetlands are essential to many long‐distance migrant waterbirds and are the main sites in China for wintering geese as “survival habitat” (e.g., Alerstam & Högstedt, 1982) to overwinter and prepare body stores for spring migration and investment in reproduction (Warnock, 2010) on their breeding areas which extend from the high Arctic Russia to lower latitude Mongolia and northeast China (Cao, Barter, & Lei, 2008; Cao, Zhang, Barter, & Lei, 2010). Rapid economic growth in the YRf has caused major ecological degradation and loss of wetlands, in part caused by the construction of many dams, including the three gorges dam (TGD) in 2003, the largest hydroelectric dam in the world (Fang et al., 2006; Guo, Hu, Zhang, & Feng, 2012; Wu & Liu, 2015; Wu et al., 2004; Xie, 2003). The highly biologically productive YRf wetlands provide ecosystem services to millions of people as well as wildlife in the form of food and water supply, transport, and mineral resources (Finlayson, Harris, McCartney, Lew, & Zhang, 2010; Fox et al., 2011); hence, there is a need to balance continuing development pressures with the cost to natural systems and the humans who rely upon them.

Poyang Lake is the largest lake in the YRf, supporting hundreds of thousands of migratory waterbirds in winter. Twenty‐nine waterbird species occur in numbers exceeding 1% of their global population, including almost all of the world's population of Siberian cranes *Grus leucogetanus*, oriental storks *Ciconia boyciana*, and swan geese *Anser cygnoides* (Ji et al., 2007). Despite protection through the establishment of Poyang Lake and Nanjishan National Nature reserves in 1983 and 2008, the extent of Poyang wetlands has significantly declined since 2000, attributed to changes in precipitation (Wu & Liu, 2015), TGD (Zhang et al., 2014) and extensive sand mining (Lai et al., 2014). To combat this wetland loss, the Jiangxi Provincial Government submitted a Poyang Lake water control project proposal (hereafter “Poyang Dam”) in 2012 to retain high winter water levels within the lake (about 10–12 m Wusong datum during November–March, *c*. 4 m higher than the average winter levels) to protect the lake ecosystem from further deterioration (Zhang et al., 2014). The proposal has raised scientific concerns because it will maintain constantly high water levels throughout the dry season (e.g., Jiao, 2009), increase inundation frequencies in late autumn and winter, submerge all current shallows and flats, and affect water quality through reduced water velocity (Lai, Wang, & Li, 2016). Wintering YRf waterbird distributions are determined by inundation areas and water quality (Q. Jia, unpublished data). The extent, timing, and duration of inundation are also critical for macrophytic growth as a source of waterbird food. Delayed flooding shortens the macrophyte growing period, reducing biomass (Cao et al., 2011; Wang, Lee, & Cheng, 2005; Zhang et al., 2011), and may result in declining numbers of waterbirds as already shown in major river basins in Australia (Kingsford, 2000; Kingsford, Bino, & Porter, 2017; Kingsford & Thomas, 2004). Because of these predicted consequences of Poyang Dam on waterbirds, we investigated the current changes in spatial distributions of two goose species in the lake in relation to the availability of their contrasting food resources.

Migratory geese in China have declined in the past 50 years (Cao et al., 2008; Zhang, Jia, Prins, Cao, & de Boer, 2015). Their decline has been attributed to their confinement to natural habitats in China (Yu et al., 2017), which are currently subject to serious loss and degradation (Fang et al., 2006; Jia et al., 2016). In contrast to their conspecific elsewhere, geese in China are missing out on the energetic benefits of feeding on agricultural land (Fox & Abraham, 2017), probably due to human activities and hunting (Yu et al., 2017). Failure of individuals to satisfy their energetic requirements on wintering sites may have immediate survival consequences, but also affect subsequent survival and reproduction (“carryover effects”) (Harrison, Blount, Inger, Norris, & Bearhop, 2011) such that habitat loss impacts demography. Wintering greater white‐fronted geese *Anser albifrons* (hereafter white‐fronted geese) and swan geese use Poyang Lake extensively during October–March and have also declined at the site during the last decade (Zhang et al., 2015). White‐fronted geese graze short graminoid swards, which are highest in protein and lowest in structural carbohydrates with initial onset of growth (Cadieux, Gauthier, Hughes, & Burger, 2005; Heuerman, 2007; Zhang, Prins, Cao, Zhao, & de Boer, 2016). Their diet is dominated by *Carex* spp. (Zhang & Lu, 1999; Zhao, Cao, Klaassen, Zhang, & Fox, 2015) which grow rapidly in autumn as water table recession exposes bare substrates (de Leeuw et al., 2006). Their numbers have declined in China since the late 1980s, especially at East Dongting Lake, where loss of suitable sedge swards has been caused by earlier water table recession following the construction of the TGD (Zhao, Cong, Barter, Fox, & Cao, 2012). Increases at Anhui lakes were associated with slower water level recession rates that favor growth of short *Carex* (Zhao et al., 2012). We therefore predict that the construction of Poyang Dam will reduce the extensive white‐fronted geese feeding areas, forcing them to use other (suboptimal) feeding areas or move elsewhere.

The larger swan geese are tuber‐feeding specialists, extracting storage organs of submerged macrophytes, mainly tubers of *Vallisneria* spp., by grubbing in substrates under shallow water and soft mud made available by winter water level recession (e.g., Barzen, Engels, Burnham, Harris, & Wu, 2009; Fox et al., 2008). *Vallisneria* tubers are energy‐rich and low in fiber and ash (Lu & Zhang, 1996), making associated handling time profitable at high rhizome densities (Fox et al., 2008). Swan geese are highly vulnerable to hydrological change. High water levels put tubers beyond their reach, and too rapid water level recession desiccates substrates, rendering food inaccessible (Fox et al., 2008). Swan geese were formerly abundant throughout the YRf but are now confined to Poyang Lake due to the loss of submerged macrophytes, mainly *Vallisneria* spp., throughout the YRf (Fang et al., 2006; Fox et al., 2011; Jia et al., 2016; Zhang et al., 2011). Elevated water levels post‐Poyang Dam construction will affect swan geese accessibility to tubers and food abundance, as *Vallisneria* tuber production is dependent on variable water conditions (Barzen, 2008).

This study compares the winter distribution of the two specialist goose species (white‐fronted and swan geese) in Poyang Lake, in relation to water levels and remotely sensed vegetation types. The dynamic lake hydrology provided contrasting water levels in consecutive winters (continuous water level recession in 2015 and consistently high water levels in 2016). Using GPS loggers, we monitored the movements of white‐fronted and swan geese within Poyang Lake across both years to compare their habitat selection under contrasting water level regimes. Our study will provide information on the likely responses of waterbirds to the construction of the proposed Poyang Dam and will contribute to making knowledge‐based conservation decisions on the management of future water levels.

## 2 MATERIALS AND METHODS

### 2.1 Study area

Poyang Lake (28º22′–29º45′N, 115º47′–116º45′E) is located in the mid‐to‐lower reaches of the Yangtze River. It has a humid subtropical climate, and water levels vary between 5 and 22 m (measured at Wu Song) throughout the year dependent on seasonal precipitation and Yangtze water levels (Lai et al., 2016). The lake area can thus vary from over 3,500 km^2^ in the wet (summer) season to <1,000 km^2^ during the (winter) dry season (Feng et al., 2012; Tan, Guo, & Wang, 2013). Winter water levels throughout the lake are highly variable due to its varied benthic topography, exposing a complex mosaic of extensive mud flats, grasslands, sedge meadows, and seasonal wetlands.

### 2.2 Vegetation map generation

**Figure 1:**
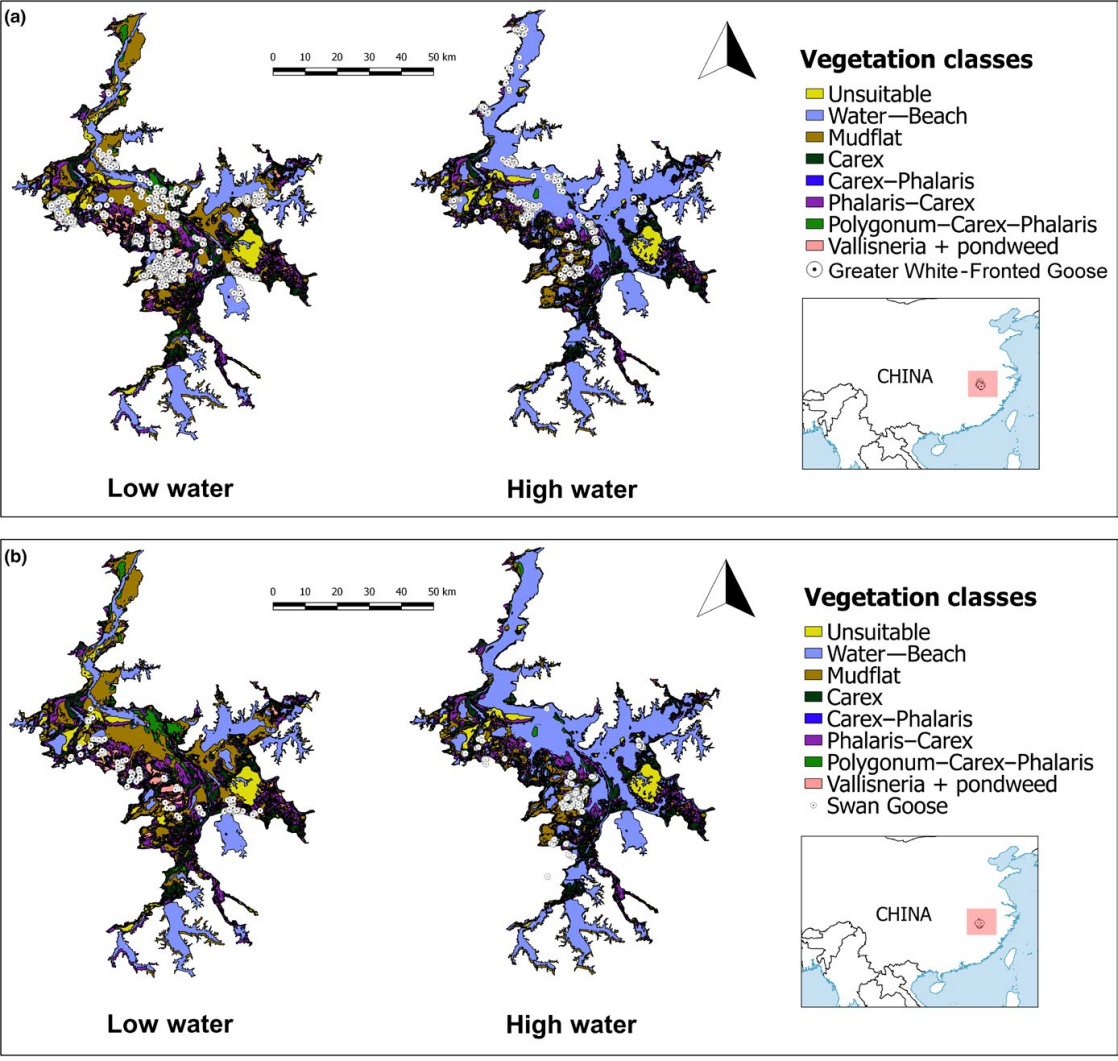
Vegetation maps of Poyang Lake under low (7.72 m) and high (12.28 m) water levels prepared from satellite images taken on 13 February 2015 and 8 February 2016, respectively. White circles depict GPS positions of greater white‐fronted geese *Anser albifrons* (a) and swan geese *Anser cygnoides* (b) in Poyang Lake (for details of map preparation, see the Methods section in main text and Appendix S1). Inset map bottom right shows the location of Poyang Lake (red square) within China

Vegetation maps of Poyang Lake were prepared using satellite images from HJ‐1 (China Centre for Resources Satellite Data and Application [http://www.cresda.com/EN/]) to represent the two water level scenarios. Satellite images were selected based on a combination of desired water level (low and high), and availability of good‐quality images for vegetation classification (see full details on the vegetation classification method in Appendix S1). We selected specific high water levels similar to those predicted in winter following the construction of Poyang Dam (stabilized at 11–12 m). These criteria resulted in images taken for vegetation classification on 13 February 2015 (water level recession scenario, 7.72 m) and 8 February 2016 (high‐water‐level scenario, 12.28 m; Figure 1). The initial vegetation map was categorized into 16 different classifications (Appendix S1). To avoid overfitting our statistical models with too many predictors, we combined classifications according to biological importance, phenology, and function and used a classification system of eight vegetation classes: *Carex*,* mixed Carex*,* mudflats*,* mixed Phalaris*,* Polygonum*,* open water*,* Vallisneria*, and *unsuitable habitat* (see details of each class in Table 1, Figure 1, and Appendix S1). In addition, we defined *water edge* as a separate “vegetation” class. This physical ecotone is a critical zone, naturally moving unpredictably in space and time, successively exposing inaccessible food resources concealed under the water and substrates for plant growth. The vegetation maps are limited in capturing the extent of this zone as it does not constitute a specific “vegetation” as such, but which does potentially straddle any of the defined classes. Because of its dynamic nature, it also lacks sharp defining boundaries. We defined *water edge* as a buffer zone 100 m to each side of the edge of imagery‐defined *open water* polygons.

**Table 1:** Wetland vegetation classifications at Poyang Lake based on interpretation of satellite images used in this study for habitat selection

Vegetation class	Details	Area (km^2^) in February 2015 (water level 7.72)	Area (km^2^) in February 2016 (water level 12.28)
*Carex*	Uniform area of C*arex* spp.	364.77	337.3
*Mixed Carex*	Dominant *Carex* mixed with *Phalaris arundinacea*	2.49	0.5
*Mudflat*	Mudflats	1066.9	551.6
*Mixed Phalaris*	Dominant *P. arundinacea* mixed with *Carex* spp.	741.83	584.4
*Polygonum*	Integration of habitats that include single stands of *Polygonum hydropiper* and habitat which is dominated by *Polygonum* mixed with *Carex* and *P. arundinacea*	148.4	54.9
*Open water*	Open water and sandy beaches and banks	920.85	1893.8
*Vallisneria*	Dominated by submergent *Vallisneria* mixed with *Potamogeton*	116.72	12.5
*Unsuitable*	Combination of habitats that were unsuitable for foraging geese and include reed, forest land, areas that are dominated by reed mixed with *Carex* and *P. arundinacea*, other *Cyperacean*‐dominated, areas dominated by *Artemisia* mixed with *P. arundinacea*, paddy fields, and artificial islands	277.94	205
*Water edge*	A buffer zone 100 m to each side of the edge of *open water* polygons	587.29	860.09

Full classification method is detailed in Appendix S1.

### 2.3 GPS geese tracking

We fitted 36 individuals white‐fronted geese and 14 swan geese with Global Positioning System (GPS) loggers to track their winter movements in Poyang Lake (Figure 1; GPS loggers and catching techniques are described in Appendix S2). We limited our analysis of individual goose distributions in time to correspond with the water level conditions in our vegetation maps (2015: 7–8 m; and 2016: 12–13 m). This restriction resulted in analyzing goose positions during the periods: 1–22 February 2015 (representing low water levels following recession, based on positions from 12 white‐fronted geese and four swan geese individuals) and 1–9 February 2016 (after consistently high water levels, 24 white‐fronted geese and 13 swan geese individuals).

Geese forage mainly during daylight and roost at night, primarily on water to avoid predation (e.g., Fox et al., 2008; Lu & Zhang, 1996), but do also feed by night (Lane & Hassall, 1996; Nolet, Bevan, Klaassen, Langevoord, & Van der Heijden, 2002; Tinkler, Montgomery, & Elwood, 2009; Ydenberg, Prins, & Van Dijk, 1984; Zhao et al., 2015). We therefore included day and night positions in the final analysis but also ran the models excluding nighttime positions to test for potential night time effects.

### 2.4 Elevation

The elevation (i.e., height above sea level) of feeding areas in wetlands contributes to determining feeding accessibility through the exposure of recessional grasslands (Zhang et al., 2016). We therefore also considered elevation in our models, using the catchment digital elevation model (DEM) from the National Geomatics Centre of China (Li, Zhang, Yao, Werner, & Li, 2013). We overlaid geese positions on the corresponding vegetation and elevation maps to extract these details at each position.

### 2.5 Statistical methods

To test whether geese selected a specific vegetation class, we developed a resource selection model by comparing the vegetation class and elevation at known GPS locations (“used”) with randomly selected points within the lake (“available”) in a ratio of three random points to every used point. Each set of random points was labeled with a corresponding ID from a tracked individual. We used binomial generalized mixed‐effect models (GLMMs) with a logit link and bird ID as random effect.

*Water edge* class was used as a binary predictor and coded 1 and 0 if the points fell within or outside the buffer, respectively. We did not include it as another vegetation class due to the high collinearity expected with other vegetation classes which lay on the water edge.

The raster layer used to extract elevation values showed that the vast majority of the lake fell within the range of 5–25 m elevation with extreme outliers in one area of deep, fast‐flowing water (−20 m) and an artificial island (123 m; Fig. S1). As both areas offered no foraging habitat for geese and geese were never observed in these areas, we restricted the selection of random points to elevations of 5–25 m to reduce variability in this predictor. Otherwise, random points were constrained within the physical boundary of the lake as shown on the vegetation maps (Figure 1).

We expected higher goose densities at intermediate elevations (i.e., a dome‐shaped curve), because at low elevation, plants have not started to grow after water recession, and in high elevation, vegetation growth is further advanced, making sward lengths too long for goose grazing (de Leeuw et al., 2006; Si et al., 2011; Zhang et al., 2016). We therefore included both elevation and elevation^2^ in our GLMMs. Our final full model included vegetation classes, water edge, elevation, and elevation^2^ as explanatory variables of geese densities in Poyang Lake. The unsuitable vegetation class was taken to be the reference category (see Table 1 for details of vegetation classes). We ran a series of competing models with increasing numbers of predictors from vegetation only to a full model containing all predictors using the *glmer* function in the lme4 R package. Model selection was undertaken by ranking AICc values and selecting the top performing model (Appendices S3 and S4). All analysis was carried out in the R programming environment version 3.0.3 (R Core Team 2014).

## 3 RESULTS

### 3.1 Vegetation class changes between years

The extent of each vegetation class at Poyang Lake varied considerably between the two winters (Figure 1; Table 1), with the greatest change in vegetation class area between low (2015) and high water level (2016) observed in *Vallisneria* (loss of 89.3%), mudflats (loss of 48.3%), open water (increased by 51.4%), *Polygonum* (loss of 63.1%), and water edge (increased by 31.7%).

### 3.2 Greater white‐fronted geese

#### 3.2.1 Vegetation class use

**Figure 2:**
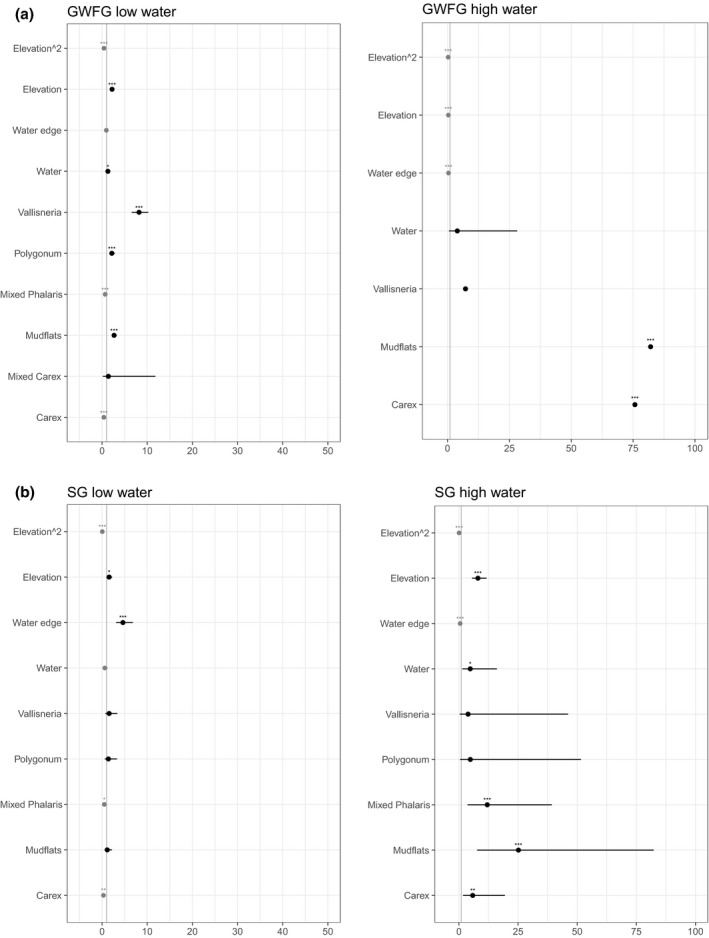
Odds ratios of fixed effect in generalized mixed‐effect models considering individual use of vegetation type, water edge, elevation, and elevation^2^ in (a) greater white‐fronted geese (GWFG) and (b) swan geese (SG). Black symbols depict positive effect, and gray symbols depict negative effect. Significant results are marked with asterisks (*** indicates *p*‐value <.001, ** indicates *p*‐value <.05, and * indicates *p*‐value = .05). Due to high collinearity of the grass classes in the model of GWFG during high water, “Carex” includes also *Phalaris* mix and *Polygonum*. See main text for details

During low water in 2015, white‐fronted geese significantly selected mudflats (*p *<* *.001), *Vallisneria* (*p *<* *.001), water (*p *<* *.01), and *Polygonum* (*p *<* *.001) relatively to the unsuitable areas. The *Polygonum* was not significant selected after exclusion of night positions from the model (results not shown), implying that it is used for roosting only. The negative correlation between white‐fronted geese distribution and *Carex* (*p *<* *.001) and mixed *Phalaris* (*p *<* *.001) suggested avoidance of longer established stands of these species (Figure 2).

During high water levels in 2016, the model resulted in high collinearity of the different grass classes (*Carex*, mixed *Phalaris*, and *Polygonum*), probably because the different vegetation classes have similar patterns of influence on geese distribution in this water level, which cannot be separated. We therefore combined the three grass vegetation classes (all containing *Carex*) into one “*Carex*” class and run the model again with only four vegetation classes. The model resulted in white‐fronted geese distribution significantly and strongly positively correlated with *Carex* (*p *<* *.001) and mudflats (*p *<* *.001) and negatively correlated with the water edge zone (Figure 2).

#### 3.2.2 Elevation

During low water levels, significant positive correlations were found between white‐fronted geese distribution and elevation (*p *<* *.001). During high water, goose distribution was negatively correlated with elevation (*p *<* *.001). A significant negative correlation with elevation^2^ in both scenarios (*p *<* *.001) suggested a dome‐shaped curve with highest goose numbers at intermediate elevations (Figure 2).

### 3.3 Swan goose

#### 3.3.1 Vegetation use

At low water levels in 2015, there was a positive significant correlation only between swan geese abundance and water edge (*p *<* *.01), and significant negative correlations between swan geese abundance and *Carex* (*p *<* *.05) and mixed *Phalaris* (*p *<* *.05) relative to unsuitable areas. At high water levels in 2016, there were significant positive correlations between swan geese abundance and *Carex* (*p *<* *.01), mudflats (*p *<* *.001), mixed *Phalaris* (*p *<* *.001), and open water (*p *<* *.05) and a negative correlation with the water edge zone (Figure 2). The positive correlation with open water area was not significant when we excluded nighttime positions (not shown), implying its use for night roosting.

#### 3.3.2 Elevation

We found significant positive correlations between swan geese distribution and elevation (*p *<* *.001) and a negative correlation with elevation^2^ (*p *<* *.001) in both low‐ and high‐water‐level periods (Figure 2), suggesting a dome‐shaped curve in both scenarios.

## 4 DISCUSSION

Our study showed that during two successive winters, the two studied goose species selected radically different plant communities as a result of major difference in water table behavior at the site. During 2015, with normal hydrological conditions of high summer water levels followed by gradual recession, the successively exposed mudflats and water edge of Poyang Lake were extensively used by the two studied goose species. White‐fronted geese exploited the fresh short growth of nutritious *Carex* and *Phalaris* as the bare substrate was exposed to support early graminoid growth accessible to the grazer species and swan geese exclusively selected the water edge at low water levels (and avoided *Carex* and mixed *Phalaris*). For swan geese, the water edge is a critical zone, moving unpredictably in space and time, exposing hidden food resources formerly inaccessible below deeper water. For this reason, this zone gathers vast numbers of other feeding waterbirds such as shorebirds, geese, cranes, and swans. In contrast, during the 2016 winter when high water levels were retained, white‐fronted and swan geese were forced to feed elsewhere, including *Carex* and *Phalaris arundinacea* habitats, although these longer established vegetations are known to be less energetically profitable for the geese. Although swan geese have been observed grazing on *Carex* and *P. arundinacea* instead of feeding on tubers (*Vallisneria*), there is good evidence that when accessible, swan geese are tuber‐consuming specialists because of the high energy content and digestibility of these underground storage organs (Fox et al., 2011; Zhang et al., 2011).

These findings have implications for the planned Poyang Dam, which is expected to affect the Poyang Lake ecosystem hydrologically, replacing the natural seasonal cycle of high summer water levels, followed by gradual water table recession to low water levels in late winter, with sustained high water levels throughout most of the winter. This will deprive the system of the normal dynamics patterns of gradual water level recession which sequentially expose bare substrate areas that offer such rich food resources for waterbirds.

The differences between low and high water levels substantially reduced the extent of optimal feeding areas available to both white‐fronted and swan geese throughout the winter, forcing them to feed on *Carex* and *Phalaris* stands where the swards were seasonally longer established and therefore of higher graminoid biomass (these vegetation classes were avoided during low water level by both species; Figure 2). Plant quality generally decreases with growing season and increased sward height (Van der Wal et al., 2000) due to increased fiber content and reduced nitrogen content (Gekara, Prigge, Bryan, Nestor, & Seidel, 2005; Prop & Vulink, 1992). Hence, taller swards are generally less energetically and nutritionally profitable and less digestible to geese (Hassall, Riddington, & Helden, 2001). Switching to suboptimal food in the absence of optimal feeding areas will likely reduce the ability of geese to achieve sufficient energy for adequate winter maintenance, especially during periods of shortest daylight and coldest temperature (e.g., Owen, Wells, & Black, 1992; Wang, Fox, Cong, & Cao, 2013), which may affect winter survival. Furthermore, failure to accumulate sufficient fat stores in preparation for migration back to the breeding grounds may carry over to affect survival and reproduction at a later stage (Aharon‐Rotman, Bauer, & Klaassen, 2016; Harrison et al., 2013; Inger et al., 2010; Morrissette, Bêty, Gauthier, Reed, & Lefebvre, 2010). This was evident in one of two springs, when lack of food resulted in lesser white‐fronted geese *Anser erythropus* departing their YRf wintering grounds with significantly less fat stores than in a year with abundant food resources (Wang et al., 2013).

In contrast to continuous high water levels, high water in summer followed by slow drawdown rates potentially provides more dynamism and greater areas of available food resources for waterbirds for longer periods throughout the season. The slow recession maintains wet surface conditions, allowing feeding by gleaners of organisms living in the exposed substrate as well as supporting subsequent growth of graminoid swards for other communities as well as grazing geese (Zhao et al., 2012). Too rapid water level recession dries out the substrates, rendering food inaccessible for species that dig for tubers and invertebrates, forcing swan geese to resort to less efficient grazing of more mature graminoid swards (Fox et al., 2008).

**Figure 3:**
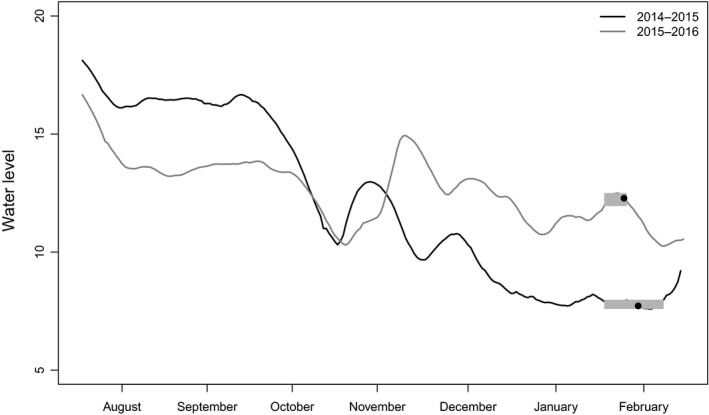
Water level changes (measured at Wu Song) from summer to winter in 2014–2015 and 2015–2016. Goose distributions in this study were analyzed during February 2015 and 2016 as low‐ and high‐water‐level scenarios, respectively. Gray rectangles depict timing of geese tracking in each winter, and black dots inside the rectangles depict the day in which satellite images were taken for vegetation classifications (13 February 2015 and 8 February 2016; see Figure 1)

Water levels in Poyang Lake prior to our study period in 2015 (low water levels) were high during summer (August water level just >18 m), gradually falling in late September, exposing new recessional grasslands (Figure 3). In 2016 (high water levels), the water level did not change significantly between summer and winter, greatly restricting the extent of newly exposed areas and fresh plant growth. For this reason, swan geese, which exclusively used the water edge in low water in 2015, avoided this zone during high water levels in 2016, probably because it was more static than in previous years and was therefore not the source of food exposed by water level recession. This contrast in water level regime between the 2 years may also explain the increased densities of white‐fronted and swan geese with elevation in 2015 (with highest abundance in medium elevation), as they exploited newly exposed feeding areas. Swan geese showed a positive correlation with elevation also during high water levels, possibly because they exploited new tuber resources at higher elevations as these were exposed in bare mud and shallow water over the winter (Barzen, 2008).

Tracking two key geese species, with contrasting feeding behaviors, enabled some generalization of our results to other waterbird species with similar feeding behavior such as cranes, shorebirds, ducks, and swans, which are affected by water level change in similar ways. Loss of submerged vegetation (or reduced accessibility to these food types) has already affected waterbirds along the YRf. For example, the loss of tuber‐producing *Vallisneria* at Shengjin Lake National Nature Reserve resulted in loss of wintering swan geese. Additionally, hooded cranes *Grus monacha* switched from feeding on tubers around the lake edge to split rice in lakeside paddies (Zhou, Zhou, Chen, Xu, & Cheng, 2009) and tundra swans *Cygnus columbianus* decreased at the site during the same period (Cheng, Cao, Barter, & Xu, 2009; Fox et al., 2011; Zhang et al., 2011). At the same time, loss of mudflats along the Yellow Sea is affecting migratory shorebirds which heavily rely on these mudflats for resting and fueling when flying between wintering and breeding sites (Studds et al., 2017).

We should be cautious about overinterpreting results from vegetation maps based on two sets of satellite images in 2015 and 2016. Firstly, as Poyang Lake water levels are highly dynamic, the exact boundaries of the vegetation classification polygons are difficult to capture. For this reason, some classifications on the edge of the water may change rapidly. We addressed this issue by creating the “water edge” variable which gathers an important area for foraging waterbirds. Secondly, the satellite images failed to capture new growth of short graminoid swards on mudflats because of the low reflectance at initial levels of biomass. The interpretation of these results therefore required extensive field experience and prior knowledge. In this way, the use of areas classified as *Vallisneria* by white‐fronted geese at low water levels was probably also due to geese grazing new *Carex* swards growing rapidly on very recently exposed mud before biomass had accumulated sufficiently to achieve sufficient reflectance to be evident on the imagery. *Vallisneria* tubers commonly occur in substrates later covered in dense *Carex* beds following water level recession, long after the autumn senescence of aboveground parts of *Vallisneria*. The lack of selection for *Vallisneria* at high water levels supports this assertion, when these communities were deeply flooded and offered no opportunity for white‐fronted geese to feed on the fresh growth of graminoids.

Poyang Dam was proposed to address the degradation of Poyang Lake and protect it from further wetland loss. However, substitution of a natural wet–dry cycle with annual permanent flooding will have lasting ecological effects on wildlife and human interests. In Australia, for example, prolonged flooding and disturbance to the wet–dry cycle led to seven species of macroinvertebrates disappearing from Lake Pedder (McComb & Lake, 1990), declines in the population of multiple waterbird species in major river basins in southeast Australia (Kingsford, 2000; Kingsford & Thomas, 2004), and high turbidity which destroyed submerged wetland vegetation in Lake Mokoan (Casanova, 1999). TGD has already radically affected sedimentation regimes in Poyang Lake which have almost certainly affected macrophyte distribution and productivity, through reduced summer water level at Hukou, the mouth of Poyang Lake which caused prolonged duration of turbidity (Zhou et al., 2016).

Based on the results of our study, it is evident that to maintain numbers of waterbirds, water levels in Poyang Lake should be allowed to fluctuate as naturally as possible, with highest water levels during summer, allowed to gradually recede through autumn to provide vital resources for waterbird species from a range of different feeding guilds. Poyang Lake is globally important site for many waterbird species, as well as providing ecosystem services to local people around the lake (Finlayson et al., 2010). It is therefore essential that any artificial solution to stem the current loss of wetland area needs to be subject to adequate environmental impact assessment and take into account the natural fluctuations that maximize productivity in any effective future management. Through carefully managed intervention in water control throughout the YRf, meeting strict environmental criteria, these hydrological requirements can be achieved with adequate consideration of human and wildlife needs.

## CONFLICT OF INTEREST

None declared.

## AUTHOR CONTRIBUTIONS

LC, YA‐R, and ADF conceived the ideas and designed methodology; HY, XW, SY, LC, and ZX collected the data; ZY and ZZ produced the vegetation maps; and JFM and YA‐R analyzed the data. YA‐R led the writing of the manuscript, with contributions from ADF. All authors contributed critically to the drafts and gave final approval for publication.
