# Supplementary material for: Water level affects availability of optimal feeding habitats for threatened migratory waterbirds
Source: Ecol Evol. 2017 Nov 7;7(23):10440–50. doi: 10.1002/ece3.3566 (PMC5723607; doi:10.1002/ece3.3566)
Supplement: Supplementary file 1 [file ECE3-7-10440-s001.docx]

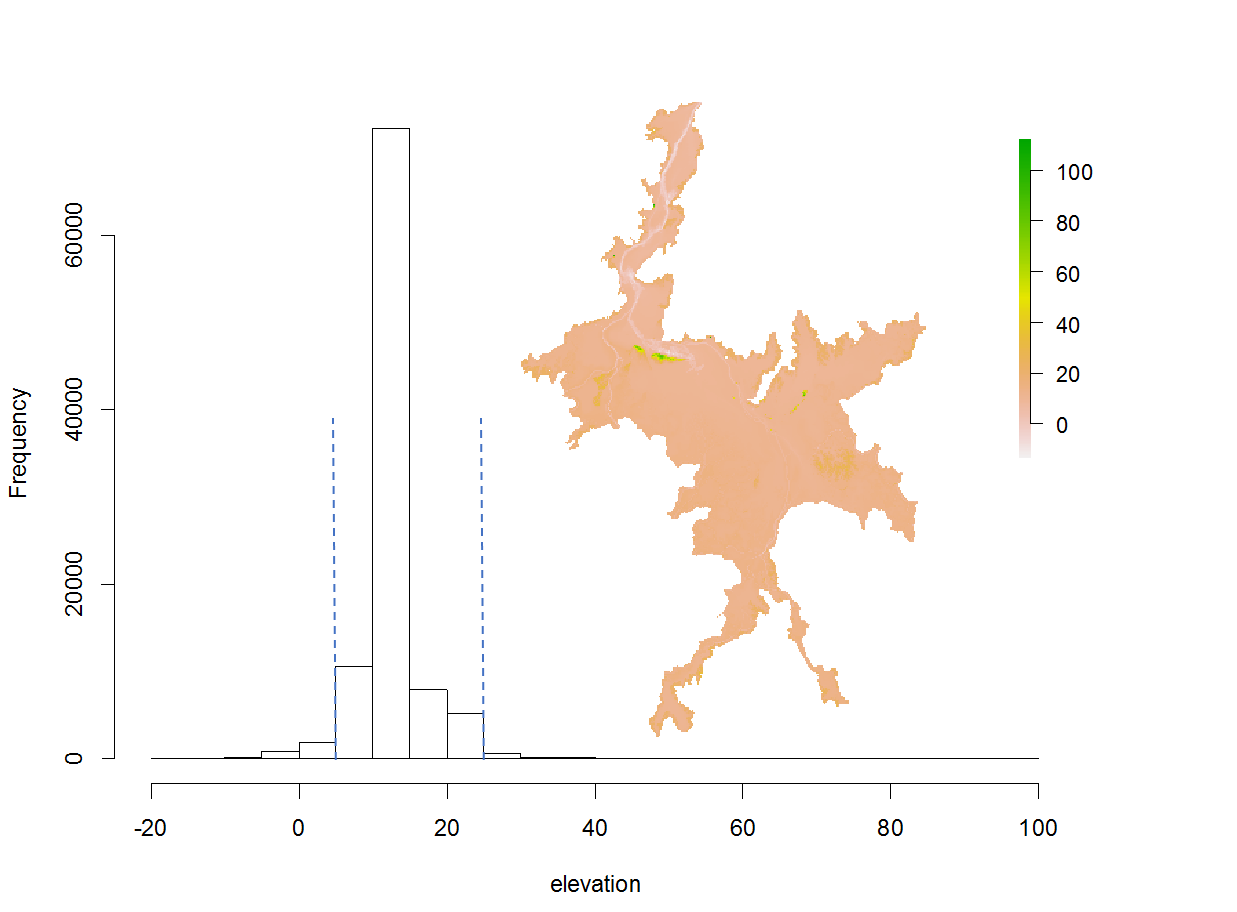
**Fig. S1.** Elevation values in Poyang Lake based on Digital Elevation Model (DEM), showing in histogram and a map illustrating the areas of extreme values. Blue dashed lines depict where we truncated the elevation selection for the analysis in the study.
