# Supplementary material for: Water level affects availability of optimal feeding habitats for threatened migratory waterbirds
Source: Ecol Evol. 2017 Nov 7;7(23):10440–50. doi: 10.1002/ece3.3566 (PMC5723607; doi:10.1002/ece3.3566)
Supplement: Supplementary file 2 [file ECE3-7-10440-s002.docx]

**Appendix S1: Vegetation classification**

Vegetation maps of Poyang Lake were prepared from HJ-1 and GF-1 data images (13 February 2015 and 8 February 2016), corresponding to periods of low and high water level, respectively (see Methods section in main text for details) and availability of clear remotely based images. The vegetation classification data for February 2015 was based on China’s domestic HJ-1 satellite remote sensing, combined with extensive ground data. The HJ-1 satellite, composed of an HJ-1A and an HJ-1B satellite, has a 700×360 km coverage with 30 m resolution. The vegetation map from February 2016 was classified using GF-1 with 16m resolution. GF-1 is equipped with two 2m panchromatic/8m multispectral cameras and four 16m wide-field imagers (WFI), which has four multispectral bands similar to HJ-1 images (blue (B1: 0.45–0.52 um), green (B2: 0.52–0.59 um), red (B3: 0.63–0.69 um), near-infrared (B4: 0.77–0.89um)). All image data are provided by the China Center for Resources Satellite Data and Application (<http://www.cresda.com/CN/>). Data pre-processing included spectral adjustment, geometric correction, and cloud removal.

Based on the purpose of the study, an optimal two levels of classification systems were proposed for the wetland images. Level I included 7 land cover types, namely forest, reed, grassland, bare inundation areas, water, cropland and artificial islands. Level II contains 16 classes, the definition of which reflects the detailed information of the main wetland vegetation types (Table S1).

Object-oriented classification was carried out using eCognition V.8.0 software. Remote sensing data were segmented into different objects, according to differences in object features of spectra, shape and texture. For the vegetation map from February 2015, an object-oriented hierarchical classification (HC) approach was applied to automatically extract Level I classes information. All the satellite data are thoroughly studied using spectral and spatial profiles to ascertain the classification rule sets of different land cover types. A decision tree classification algorithm was subsequently applied to extract information from remotely-sensed data. The ISODSTA (iterative self-organizing data analysis algorithm) module in ENVI was then used for an unsupervised classification in each of the Level I classes to acquire the 16 Level II classes. The vegetation map from February 2016 was produced by the land cover change detection method using segment similarity of spectrum vector according to a knowledge base. To rectify the errors caused by computer automatic classification, we modified the results manually by changing the classification code and in some cases manually creating a sketch patch boundary based on field survey samples and prior knowledge. The wetland cover classification results in Poyang Lake with 16 Level II classes are shown in Table S1. In order to avoid over-fitting our statistical models with too many predictors, we combined classifications according to biological importance, phenology, and function into eight vegetation classes. This combination resulted in the vegetation classes: *Carex,* mixed *Carex,* mudflats, mixed *Phalaris, Polygonum,* open water, *Vallisneria* and unsuitable habitat (see also details of each class in Table 1 and Figure 1 in the main text). The ”unsuitable” areas comprise dense, tall vegetation and were defined based on expert opinions, where during 12 years of field observations, geese were never seen feeding on these habitat types.

Because the water edge is a critical but ephemeral zone, naturally moving unpredictably in space and time, exposing hidden food under the water, we also created a ninth habitat class, ”water edge”, by making a buffer zone of 100 m to each side of the water edge (see justification in method section in the main text and Table 1).

Table S1. Wetland cover vegetation classification in Poyang Lake with two levels of classes.

| Level 1 class | Level 2  Class | Area (km^2^) in Feb. 13, 2015 | Area (km^2^) in Feb. 8, 2016 |
| --- | --- | --- | --- |
|  |  |  |  |
| Forest | Forest | 20.26 | 16.6 |
| Reed | Reed | 10.3 | 10.9 |
| Grassland | *Carex* | 364.77 | 351.3 |
|  | *Polygonum hydropiper* | 133.7 | 128.8 |
|  | *Phalaris arundinacea+Carex* | 741.83 | 631 |
|  | *Wormwood+Phalaris arundinacea* | 0.31 | 0.3 |
|  | *Reed+Carex+Phalaris arundinacea* | 19.75 | 19.7 |
|  | *Carex+Phalaris arundinacea* | 2.49 | 2.5 |
|  | *Polygonum hydropiper+Carex+Phalaris arundinacea* | 14.7 | 14.9 |
|  | Other Cyperacean dominated | 21.02 | 16.5 |
| Bare inundation areas | Mudflat | 1066.9 | 925.4 |
|  | Sandy beach | 16.03 | 18 |
| Water | *Vallisneria* | 116.72 | 14.4 |
|  | Water without submerged macrophytes | 904.82 | 1342.3 |
| Paddy field | Paddy fields | 2.03 | 2 |
| Artificial Island | Artificial island | 204.27 | 146.2 |
