# Supplementary material for: Water level affects availability of optimal feeding habitats for threatened migratory waterbirds
Source: Ecol Evol. 2017 Nov 7;7(23):10440–50. doi: 10.1002/ece3.3566 (PMC5723607; doi:10.1002/ece3.3566)
Supplement: Supplementary file 3 [file ECE3-7-10440-s003.docx]

**Appendix S2: Geese catching method and logger details**

At Nanjishan National Nature Reserve at Poyang Lake (Table S2a), geese were captured using two methods (lamping and mist-netting) to attach different types of GPS loggers (Table S2b). For lamping, teams in fast, light punts propelled by one oarsman travelled to known roosting goose areas under cover of darkness and captured geese on the water using strong (>500,000 candlepower) lamps. Geese already settled on the water to sleep at the roost site usually “froze” and did not normally move once caught in such light, enabling the catching crew punt to be steered towards the geese. The individual could be “held” within the beam until the catcher in the prow of the punt could place a long-handled hand net under the bird on the water. The bird could then be hooked upwards into the net for retrieval on board. The second method, mist-netting, required prior knowledge of the routes taken during roosting flights by geese to get from daytime feeding areas to safe night time roosts. Heavy duty wader netting (three-shelf three-metre high) was strung between strong bamboo poles for lengths of up to 50 metres in shallow water close to known roost sites, where geese were caught flying in after sunset. Teams concealed nearby would check the net lines regularly after hearing the passage of geese going to roost and quickly extract birds caught.

At Poyang National Nature Reserve (Table S2a), geese were caught using both mist-net technique and leg-noose traps. Leg-nooses were set at dawn at feeding sites and in the edge between waterbody and feeding sites. To minimize the time a bird was trapped, the nooses were monitored from a distance of 1 km once set, and nooses were retrieved at dusk on each catching attempt. At Anhui Lakes (Table S2a), geese were captured by clap nets set in shallow water, where tame ducks were confined to an area to decoy wild ducks and geese into the catching areas. The catching team watched from hides close to the catching area and quickly pulled the clap net up and over the aggregated geese from which the geese could be retrieved.

In northeast Mongolia (Table S2a), geese were captured by herding flightless adults and juveniles with boats and kayaks into corral traps (Choi *et al.* 2016).

A summary of all catching sites, periods and logger types are presented in Table S2a

**Table S2a.** Details of each of the GPS/GSM loggers fitted to Great white-fronted Geese and Swan geese in this study.

| Transmitter mounting type | Manufacturer and country of origin | Device mass (g) | Harness Material | Harness weight (g) | Abbreviation |
| --- | --- | --- | --- | --- | --- |
| Neck collar | Ecotone Telemetry, Poland | 44 | - | - | ET_NC |
| Neck collar | Hunan Global Messenger Technology Co., China | 26 | - | - | HGMT_NC |
| Backpack | Ecotone Telemetry, Poland | 31 | Teflon ribbon | 5 | ET_BP |
| Backpack | Hunan Global Messenger Technology Co., China | 27 | Custom made compound ribbon | 10 | HGMT_BP |
| Backpack | KoEco Inc., South Korea | 50 | Teflon ribbon strengthened with wire internally | 5 | KI_BP |

**Table S2b**. Catching details and logger types for each goose species used in this study (see detailed technical data on loggers in Table S2a). Body weights tabulated and used in comparisons with logger weight were obtained from Johnsgard (1978).

| Species | Year (water level) | Catch sites (number of individuals per site) | Location | Catching dates | Logger type (number of individuals) | Body weight range (kg) | Logger percentage body weight |
| --- | --- | --- | --- | --- | --- | --- | --- |
| Great white-fronted geese | 2015 (low) | Poyang Lake (12) | 29°07’N 116°06’E | 2014/12/29,2015/1/29 | HGMT_BP (8) | 1.6-2.64 | 1.0-2.3% |
|  |  |  |  | 30/1-2/2/2015 | ET_NC (4) |  |  |
|  | 2016 (high) | Anhui Lakes (6) | 30°24’N 117°04’E | 15/10/2015 | HGMT_BP (6) |  |  |
|  |  | Poyang Lake (19) | 29°07’N 116°06’E | 9-14/12/2015 | HGMT_NC (18) |  |  |
|  |  |  |  |  | KI_WT300s (1) |  |  |
| Swan geese | 2015 (low) | Eastern Mongolia Lakes (4) | 49°44’N 115°13’E | 27-29/7/2014 | ET_NC (4) | 1.97-3.96 | 0.8-2.2% |
|  | 2016 (high) | Anhui Lakes (2) | 30°24’N 117°04’E | 16/11/2015 | ET_NC (2) |  |  |
|  |  | Poyang Lake (12) | 29°07’N 116°06’E | 10-13/12/2015 | HGMT_NC (4) |  |  |
|  |  |  |  |  | KI_WT200 (4)  ET_NC (4) |  |  |

^*^Total number of individuals in each year/water level scenario includes duplicates due to individuals with multiple years of data (one individual GWFG and three individuals SG).

^**^Abbreviations- HGMT: Hunan Global Messenger Technology Co., China; ET_NC: Ecotone Telemetry, Poland; KI_BP: KoEco Inc., South Korea.

**References**

Choi, C.-Y., Lee, K.-S., Poyarkov, N.D., Park, J.-Y., Lee, H., Takekawa, J.Y., Smith, L.M., Ely, C.R., Wang, X. & Cao, L. (2016) Low Survival Rates of Swan Geese (*Anser cygnoides*) Estimated from Neck-Collar Resighting and Telemetry. *Waterbirds,* **39,** 277-286.

Johnsgard, P.A. (1978) *Ducks, geese, and swans of the world*. University of Nebraska Press Lincoln.
