# Supplementary material for: Water level affects availability of optimal feeding habitats for threatened migratory waterbirds
Source: Ecol Evol. 2017 Nov 7;7(23):10440–50. doi: 10.1002/ece3.3566 (PMC5723607; doi:10.1002/ece3.3566)
Supplement: Supplementary file 4 [file ECE3-7-10440-s004.docx]

**Appendix S3:**  Top selected models of greater white-fronted geese abundance during low (a) and high (b) water level in February 2015 and 2016, respectively. Geese abundance is based on GPS loggers positions with respect to vegetation type (VegClass), elevation and elevation^2^ (elev^2^). We ran a series of competing models with increasing numbers of predictors from vegetation only to a full model containing all predictors. Model selection was carried out by ranking AICc values and selecting the top performing model. Only top models are presented. logLik: log likelihood

Table S3a: Great White-fronted Geese in low water level

| Model | Intercept | VegClass | Water edge | elevation | elev^2^ | logLik | AICc | delta | weight |
| --- | --- | --- | --- | --- | --- | --- | --- | --- | --- |
| Global Model | -1.107 | + | -0.04623 | 0.7962 | -0.8495 | -7102.021 | 14228.1 | 0 | 1 |
| mod.1 | -2.16 | + |  | 0.3347 |  | -7624.476 | 15269 | 1040.9 | 0 |
| mod.2 | -1.687 | + |  |  |  | -7737.138 | 15492.3 | 1264.22 | 0 |
| mod.3 | -1.686 | + | -0.00997 |  |  | -7737.126 | 15494.3 | 1266.2 | 0 |

| Model | Intercept | VegClass | Water edge | elevation | elev^2^ | logLik | AICc | delta | weight |
| --- | --- | --- | --- | --- | --- | --- | --- | --- | --- |
| Global Model | -3.891 | + | -0.9248 | -1.122 | -1.356 | -2804.269 | 5626.6 | 0 | 1 |
| mod.1 | -4.867 | + |  | -0.8611 |  | -3064.801 | 6143.6 | 517.05 | 0 |
| mod.2 | -5.511 | + | -0.7933 |  |  | -3189.697 | 6393.4 | 766.85 | 0 |
| mod.3 | -5.606 | + |  |  |  | -3233.566 | 6479.1 | 852.58 | 0 |

Table S3b: Great white-fronted geese in high water level

**Appendix S4:**  Top selected models of swan geese abundance during low (a) and high (b) water level in February 2015 and 2016, respectively. Geese abundance is based on GPS loggers positions with respect to vegetation type (VegClass), elevation and elevation^2^ (elev^2^). We ran a series of competing models with increasing numbers of predictors from vegetation only to a full model containing all predictors. Model selection was carried out by ranking AICc values and selecting the top performing model. Only top models are presented. logLik: log likelihood

Table S4a: Swan geese in low water level

| Model | Intercept | VegClass | Water edge | elevation | elev^2^ | logLik | AICc | delta | weight |
| --- | --- | --- | --- | --- | --- | --- | --- | --- | --- |
| Global Model | -0.1731 | + | 1.531 | 0.4478 | -2.84 | -580.147 | 1182.5 | 0 | 1 |
| mod.1 | -1.578 | + | 1.26 |  |  | -709.978 | 1438.1 | 255.6 | 0 |
| mod.2 | -1.221 |  | 1.048 |  |  | -732.984 | 1472 | 289.49 | 0 |
| mod.3 | -1.221 |  | 1.047 | -0.007049 |  | -732.977 | 1474 | 291.49 | 0 |

Table S4b: Swan geese in high water level

| Model | Intercept | VegClass | Water edge | elevation | elev^2^ | logLik | AICc | delta | weight | |
| --- | --- | --- | --- | --- | --- | --- | --- | --- | --- | --- |
| Global Model | -2.763 | + | -0.6508 | 2.083 | -2.544 | -1072.818 | 2167.7 | 0 | 1 |  |
| mod.1 | -4.593 | + |  | 0.369 |  | -1231.621 | 2481.3 | 313.58 | 0 |  |
| mod.2 | -3.612 | + | -0.6802 |  |  | -1232.05 | 2482.2 | 314.43 | 0 |  |
| mod.3 | -3.664 | + |  |  |  | -1243.773 | 2503.6 | 335.86 | 0 |  |
